# Vitamin D monitoring and management within men's secure services

**DOI:** 10.1192/bjo.2021.885

**Published:** 2021-06-18

**Authors:** Jason Niblett, Shay-Anne Pantall, Anis Ahmed

**Affiliations:** 1 University of Birmingham, Medical School; 2 Birmingham and Solihull Mental Health NHS Foundation Trust

## Abstract

**Aims:**

To audit the investigation, identification and treatment of Vitamin D deficiency within Men's Secure Services.

**Background:**

Vitamin D and/or vitamin D deficiency has been suggested to play a role in the pathogenesis of mental illness. There is evidence that Vitamin D inadequacy is pandemic among rehabilitation patients in inpatient settings. Patients within secure hospitals are similarly considered to be at high risk, due to their limited solar exposure during often lengthy admissions. It has been suggested that these patients should be considered an ‘at-risk’ cohort, for whom Vitamin D supplementation should be routine. Men's secure services in Birmingham comprise of two medium secure units and a low secure rehabilitation unit. Here we present an audit of Vitamin D monitoring and treatment completed in 2019.

**Method:**

A three year retrospective review of electronic patient records, for all inpatients admitted within men's secure services as of 1 September 2019 (n = 188). Standards were based on the Trust accepted guidelines for management of Vitamin D deficiency.

**Result:**

Key findings included:-

- The majority of inpatients were Caucasian (43%) and African-Caribbean (24%). Ages ranged from 18 to 70, with a mean age of 39.
- Approximately two-thirds (65%) had been in hospital for over a year, of which 44% had been admitted for more than 3 years. The average length of admission was 885 days.
- Only 47% of patients had their Vitamin D level checked within the study period.
- Of those checked, 24% were tested within 1 month of admission. The mean duration between admission and Vitamin D testing was 464 days.
- Results ranged from 10.3 to 118.5nmol/L. A high rate of Vitamin D deficiency was identified (54%), whilst a further 16% had ‘inadequate’ levels.
- 23% of those identified as requiring treatment did not receive any supplementation, whilst 59% of those with sufficient Vitamin D were prescribed treatment.
- Only 48% had their levels rechecked following treatment; of these, only 59% now had an adequate Vitamin D status.

**Conclusion:**

This audit demonstrates limited Vitamin D monitoring within male forensic inpatients. There was a high prevalence of Vitamin D insufficiency in this population, yet a substantial proportion of patients with identified deficiency were not prescribed any treatment. Ongoing monitoring and review of treatment effectiveness was poor. We argue that more consideration should be given to this population, with robust guidelines introduced for the treatment of this specific ‘at-risk group’.

